# A Clinical Study of Urine Amino Acids in Children with Autism Spectrum Disorder

**DOI:** 10.3390/life14050629

**Published:** 2024-05-15

**Authors:** Cătălina Mihaela Anastasescu, Veronica Gheorman, Florica Popescu, Mioara Desdemona Stepan, Eugen Cristi Stoicănescu, Victor Gheorman, Ion Udriștoiu

**Affiliations:** 1 Hospital of Neuropsychiatry Craiova, Children Mental Health Center, 200349 Craiova, Romania; catalina_tocea@yahoo.com; 2 Department 3 Medical Semiology, University of Medicine and Pharmacy of Craiova, 200349 Craiova, Romania; 3 Pharmacology Department, University of Medicine and Pharmacy of Craiova, 200349 Craiova, Romania; prof_floricapopescu@hotmail.com; 4 Department of Infant Care-Pediatrics-Neonatology, University of Medicine and Pharmacy of Craiova, 200349 Craiova, Romania; 5 Pediatry Department, Emergency Clinical Hospital Râmnicu Vâlcea, 200300 Râmnicu Vâlcea, Romania; prof.dr.stoicanescu@gmail.com; 6 Department of Psychiatry, University of Medicine and Pharmacy of Craiova, 200349 Craiova, Romania; victor.gheorman@umfcv.ro (V.G.); ion.udristoiu@umfcv.ro (I.U.)

**Keywords:** urine, amino acids, autism, children

## Abstract

Amino acids are organic compounds that enter the protein structure, being involved in the proper functioning of the body. The role of amino acids in the onset of autism spectrum disorder (ASD) is yet to be established. Our aim was to identify correlations between urine amino acids and their derivatives and ASD. Methods: We designed a case–control study that consisted of 75 boys and girls, aged between 2 and 12 years. For amino acid profile, we used urine samples that were analyzed using liquid chromatography–tandem mass spectrometry (LC-MS/MS). Results: Descriptive analysis showed higher values for glutamine, hydroxyproline, tyrosine, aspartic acid, and tryptophan and lower values for serine in the autism group than in the control group. Also, we found that boys with autism had higher values than the boys in the control group for serine, threonine, and aspartic acid. For girls from both groups, we did not find statistically significant values. In terms of age groups, we found significantly higher values for histidine, threonine, valine, methionine, aspartic acid, glutamic acid, alpha amino-adipic acid, sarcosine, alanine, and beta-alanine and significantly lower values for proline for both the autism and control groups under 5 years. Conclusions: The findings of this study support the assumption that amino acids may have a role in the expression of ASD.

## 1. Introduction

Autism spectrum disorder (ASD) is known as a developmental disorder, one that is diagnosed in the early stages of life, but the reasons for its occurrence are not fully understood. It is believed that the involvement of chemicals such as amino acids in brain development may play a significant role in the onset and expression of this disorder [1,2,3,4]. Amino acids are of crucial importance in brain function, not only as metabolic intermediates and protein synthesis material, but also as mediators of inter-neuronal communication [5,6]. Peripheral concentrations of some amino acids may be correlated with central brain functions, and changes in amino acid bioavailability and/or neurotransmitter metabolism may contribute to the pathogenesis of psychiatric disorders [6]. Levels of neuroactive amino acids in body fluids (blood, urine, etc.), including glutamate, glutamine, taurine, gamma-aminobutyric acid (GABA), glycine, tryptophan, and serine have been widely studied in ASD, but results reported in the literature are inconclusive [5,6]. Current research on amino acid levels and metabolism in ASD patients has brought to light important information regarding the involvement of these molecules in their health and behavior. Abnormalities in amino acid metabolism are found to contribute to the manifestation and severity of symptoms associated with ASD. For example, a decrease in plasma levels of essential amino acids such as leucine, isoleucine, and valine has been observed [7]. These abnormalities can affect protein synthesis, an essential process in the development and functioning of the central nervous system. Tryptophan deficiency and low serotonin levels may contribute to the anxiety and aggression seen in ASD. In addition, amino acids paly an essential role in the regulation of neurotransmitters. GABA (γ-aminobutyric acid) and glutamate, two of the most important neurotransmitters in the central nervous system, are regulated by amino acid levels. Higher amount of glutamate, an excitatory amino acid, may be involved in the repetitive behaviors and communication disturbances seen in this disorder. It is revealed that ASD patients show imbalances in the levels of these neurotransmitters, suggesting a dysfunction in amino acid metabolism and a possible contribution to the specific symptoms of the disorder [8]. There is evidence that essential amino acid supplementation improves repetitive behaviors and communication deficits in children with ASD [9]. This supports the assumption that dysfunctions in amino acid metabolism may contribute to the manifestation of ASD specific behaviors.

Starting from the hypothesis that amino acids may be involved in the pathophysiology of ASD, we aimed to better understand these potentially psychopathological mechanisms in order to be able to open new therapeutic opportunities in ASD.

## 2. Materials and Methods

### 2.1. The Purpose of the Study

In our study, we aimed to analyze the values of 32 urine amino acids to determine differences between autistic children and typically developing children, as well as to assess variations according to age and sex variables. Our primary objective was to investigate whether autistic children exhibit distinct amino acid profiles compared to their typically developing counterparts. Additionally, we sought to explore potential associations between amino acid levels and demographic factors such as age and sex.

### 2.2. The Type of the Study

This is an observational, case–control study, conducted in the pediatric psychiatry clinic during 2021–2022.

### 2.3. Informed Consent

The study protocol received approval from the Ethics and Scientific Academic Deontology Commission of the University of Medicine and Pharmacy in Craiova (No. 154/24.09.2021), ensuring adherence to the principles outlined in the Declaration of Helsinki and the Code of University Ethics. This endorsement confirmed that the research procedures complied with ethical standards, in accordance with the guidelines specified in the Medical Ethics Code. All parents or legal guardians signed the informed consent for the data publication. 

### 2.4. Statistical Analysis

We operated on a Windows 10 system. The program IBM SPSS version 23 was used for the statistical analysis. MS Office Excel 2016 and MS Office Word 2016 was used for the graphs and charts. To assess age-related differences, following cluster discrimination analysis, each group was subdivided into two distinct (ANOVA, *p* < 0.0001) and homogenous (Wilk’s Lambda) subgroups: the autism patient group < 5 years, autism patient group > 5 years, control group < 5 years, and control group > 5 years.

### 2.5. Study Groups

This case–control study spanned between 2021 and 2022. Both boys and girls between 2 and 12 years old were recruited for the study. The study group consisted of 45 children diagnosed with autism, and the control group comprised 30 children without any psychiatric pathology. Participants in the study group were recruited from children evaluated and diagnosed with autism spectrum disorder (ASD) at a private medical office in Craiova, Romania, certified by the Dolj Public Health Directorate and the Romanian College of Physicians, Dolj branch, between the years 2017 and 2022.

Selection criteria were outlined in the study information form and included boys and girls aged 2–12 years that were diagnosed with an autistic disorder; children diagnosed with ASD without metabolic or genetic diseases that could impact laboratory test results; children with ASD who had not received treatment or medical interventions affecting laboratory samples for at least two months prior to collection; and children with ASD who had not taken food supplements for two months prior to laboratory sample collection. Informed consent was mandatory for inclusion. 

We excluded children with different medical disorders (including genetic or metabolic); children treated with medicines or supplements that could affect amino acid concentrations; children outside the age range of 2–12 years specified for the study; agitated children or those from whom laboratory samples could not be collected for various reasons; and children whose relatives declined to sign the acceptance form or withdrew from the study.

On the other hand, the control group consisted of volunteers selected on the same criteria of age, sex, and background from the general population. They were recruited from different schools and kindergartens in the southern area of Oltenia and voluntarily participated in this research study. For awareness of the study, an information form was used, specially created for enrollment in the control group.

**Figure 1:**
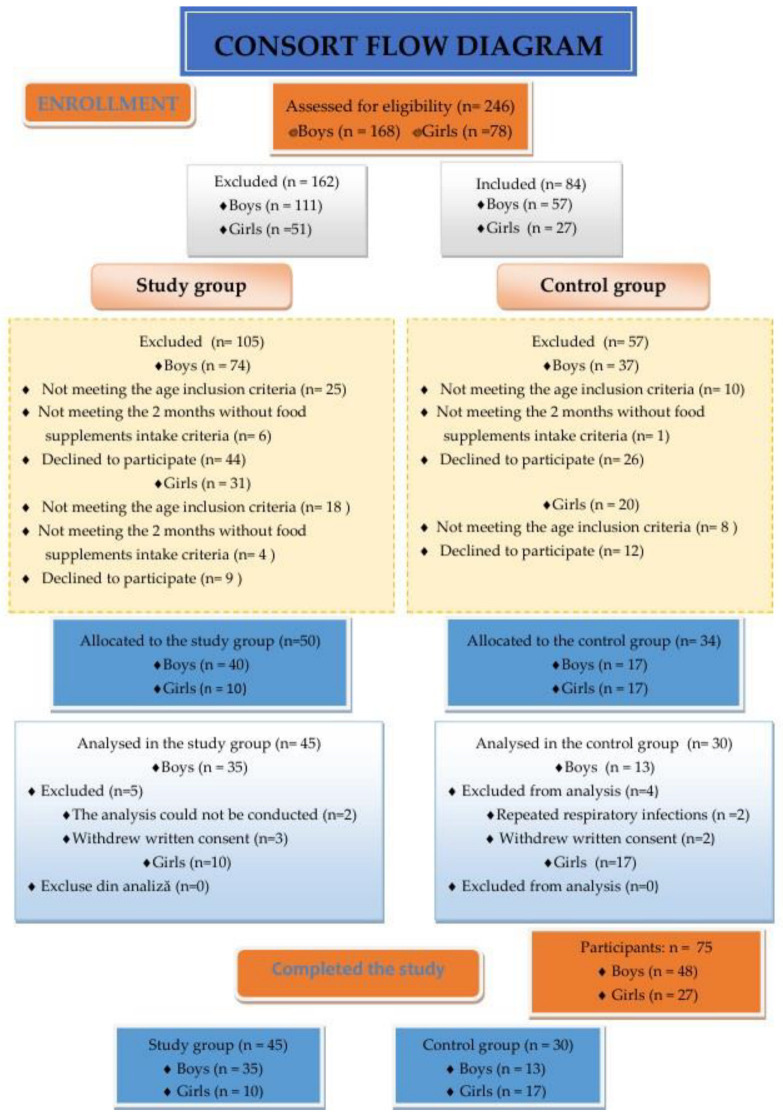
Enrolment of participants; detailed consort flow diagram.

Detailed data related to the study group and the control groups are described by the Consort Diagram as follows (Figure 1):

### 2.6. Study Cohort—Descriptive Analysis

**Figure 2:**
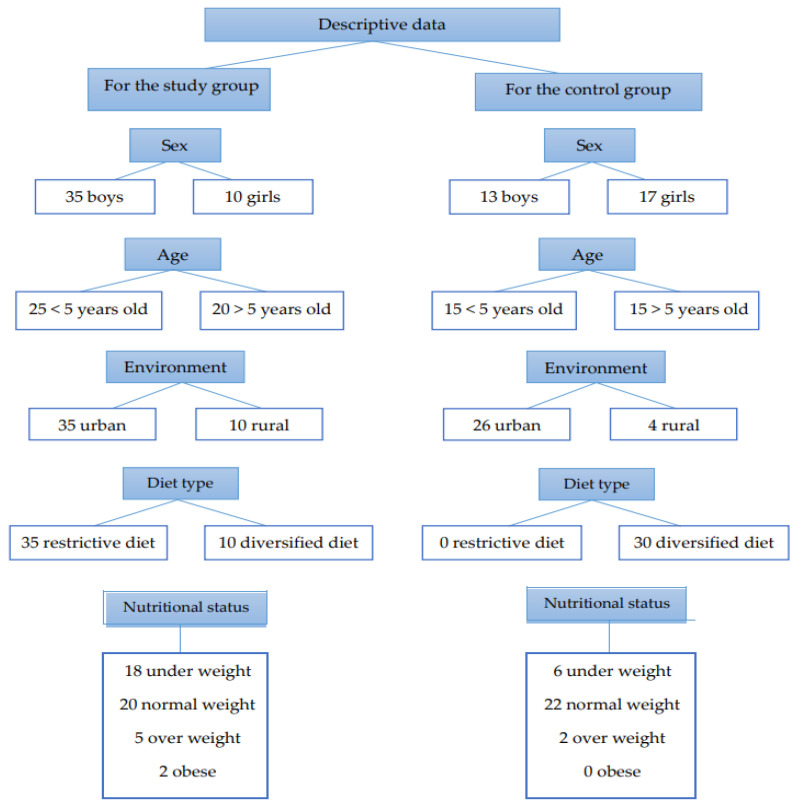
Descriptive data for the study group and the control group.

Based on gender, the study group comprised 35 boys and 10 girls, while the control group was 13 boys and 17 girls. Distribution by age consisted of 25 children under 5 years old and 20 children over 5 years old in the study group, whereas the control group included 15 children under 5 years old and 15 children over 5 years old. In terms of urban or rural origin, 35 children in the study group were from urban areas and 10 from rural areas, compared to 26 urban and 4 rural children in the control group. Regarding dietary habits, 35 children in the study group followed a restrictive diet, while 10 had a varied diet. In contrast to children in the study group, all children from the control group had a varied diet. Nutritional status varied in the study group, with 18 children classified as underweight, 20 with a normal weight, 5 children as overweight, and 2 were obese. In the control group, 23 had normal weight, 6 children were underweight, and 2 were overweight (Figure 2).

### 2.7. Collection and Testing of Laboratory Samples

Urine samples were collected between October 2021 and January 2022. Patients and volunteers were scheduled to present at the laboratory early in the morning after an overnight fasting. The recommendation was for the diet to remain usual in the 2–3 days leading up to sample collection, as well as to avoid the intake of any medication that could potentially alter the results. A minimum of 10 mL of spot urine collected in the morning was required to perform the urinary amino acid profile. Since immediate processing of the samples was not feasible and they needed to be sent to another laboratory equipped with the appropriate technology for this research component, the samples were frozen at −20 °C after the addition of a few drops of glacial acetic acid. They were then transported in dry snow for testing.

Liquid chromatography–tandem mass spectrometry (LC-MS/MS) was employed for the analysis of the amino acid profile in urine. The analysis focused on the following amino acids and amino acid derivatives, presented in alphabetical order: alpha aminoadipic acid, alpha aminobutyric acid, anserine, arginine, aspartic acid, asparagine, beta-alanine, citrulline, cystine, cystationine, glutamine, glutamic acid, glycine, histidine, homocysteine, hydroxylisine, hydroxyproline, isoleucine, leucine, lysine, methionine, methylhistidine, ornithine, phenylalanine, proline, sarcosine, serine, taurine, threonine, tryptophan, tyrosine, and valine.

We received the sample results within 12 to 15 working days after collection.

## 3. Results

### 3.1. Analysis of Amino Acids in Urine in the Study Group Compared to the Control Group

We performed a descriptive statistical analysis of the urinary amino acid profile using Student’s *t*-test for equal variances and for unequal variances, when appropriate (Table 1).

For the comparison of means of alpha-amino-adipic acid, alanine, beta-alanine, and sarcosine, we used non-parametric tests. Alpha amino-adipic acid had *p*-values of 0.53 for both the study group and control group in the Kolmogorov–Smirnov test. Also, we obtained a 0.32 *p*-value for the study group and a 0.37 *p*-value for the control group in the Shapiro–Wilk test, without statistical significance. The values for alanine and beta alanine were close and had no statistical significance. For sarcosine, we obtained a *p*-value of 0.37 for the study group and the control group using the Kolmogorov–Smirnov test. In the Shapiro–Wilk test, the results were a 0.69 *p*-value for the study group and a 0.70 *p*-value for the control group, without statistical significance.

For some of the analyzed amino acids, the comparison of means was performed using the non-parametric tests: Mann–Whitney and Wilcoxon tests (Tables S1 and S2). For hydroxylysine, anserine, cystathionine, and homocysteine, the values were lower than 1, so statistical analysis could not be performed.

**Figure 3:**
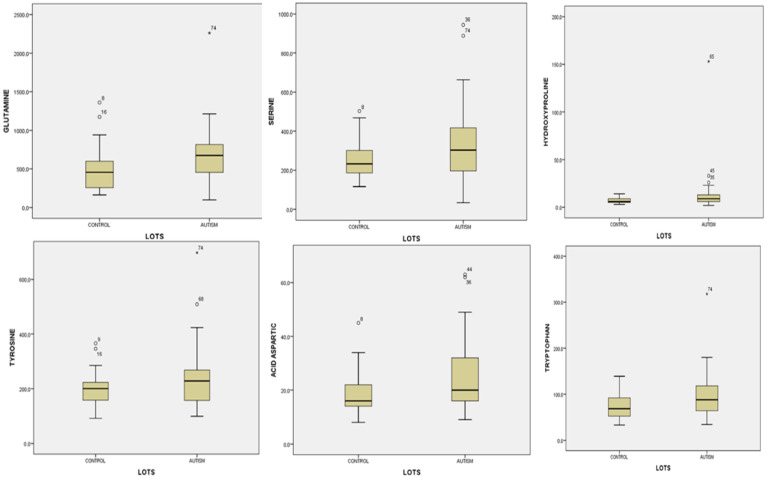
Urine level of glutamine, serine, hydroxylysine, tyrosine, aspartic acid, and tryptophan in the study group (autism) versus the control group. Cases with extreme values outside the 95% CI (outliers) are represented by circles and asterisks in the boxplot.

For glutamine in urine, we obtained statistically significant values: t = −2.191, *p =* 0.032. Children with autism had significantly higher values compared to children in the control group: 678.60 ± 362.71 versus 511.23 ± 295.45. Also, after the statistical analysis of the glutamine values for the two groups, we obtained significant values with the non-parametric Wilcoxon test: U = 447, *p =* 0.014 (Z = −2.466) (Figure 3). The values of serine in the urine for the group of children with autism were significantly lower compared to the values obtained in the control group: 259.00 ± 105.83 versus 327.93 ± 188.04, *p* = 0.047 (Figure 3). For hydroxyproline in urine, we obtained statistically significant differences between the values of the two groups: U = 493.5, *p =* 0.048. Children with autism had significantly higher values compared to children in the control group (Figure 3). For urinary tyrosine, Student’s *t*-test for unequal variances showed a statistically significant difference between the values obtained for the study group: 241.46 ± 113.05 and the control group: 198.80 ± 66.25. Tyrosine values for children with autism were higher compared to children who did not have psychiatric pathology, *p =* 0.043 (Figure 3). For aspartic acid in urine, we obtained statistically significant values with the Mann–Whitney test, *p =* 0.029, with children with autism having significantly higher values than children without psychiatric pathology: 25.24 ± 13.30 versus 19.06 ± 8.16 (Figure 3). We also obtained statistically significant values for tryptophan in urine. The children in the study group had significantly higher values compared to the children in the control group: 95.93 ± 47.69 versus 75.76 ± 29.95, *p =* 0.028 (Figure 3).

### 3.2. Statistical Analysis of Amino Acids in Urine According to Sex for the Study Group and the Control Group

We performed correlations for amino acid values according to sex. We performed the Tamhane test and Dunnett test for unequal variances (Table 2).

For urinary alpha-aminobutyric acid, the Kruskal–Wallis test for non-parametric groups showed significance across groups: *p* = 0.033, but pairwise analysis failed to detect significant differences. The same was true for hydroxyproline in urine, *p* = 0.045, but without revealing significant differences in pairwise analysis.

Although there were differences between urinary amino acid values of children with autism and the control children, we found statistical significance only for serine, threonine, and aspartic acid after using Tamhane and Dunnet tests for unequal variances (Table S3).

**Figure 4:**
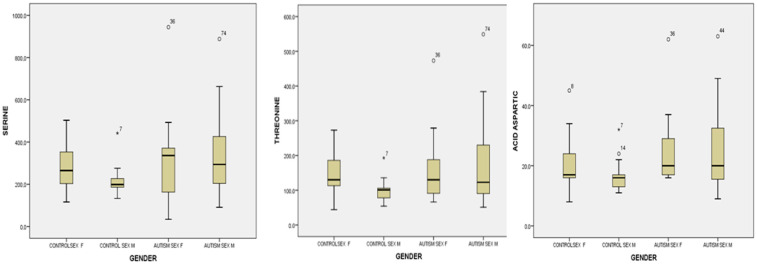
Urine serine, threonine, and aspartic acid levels by male and female sex for the control group and the study group (autism). Cases with extreme values outside the 95% CI (outliers) are represented by circles and asterisks on the boxplot.

For urinary serine, after the analysis for unequal variances (Tamhane test and Dunnet test), we found statistically significant differences between autistic boys and control boys, 322.82 ± 170.75 versus 215.61 ± 77.86, *p =* 0.028. The girls in the group of children with autism had higher values than the girls in the control group, but without statistical significance (Figure 4). For urinary threonine, boys from the group of children with autism had significantly higher values than boys from the control group: 170.40 ± 109.42 versus 100.69 ± 38.02, *p =* 0.012. The girls in the group of children with autism had higher values than the girls in the control group, but without statistical significance. For urinary aspartic acid, after performing statistical analysis for unequal variances applying Tamhane’s test and Dunnet’s test for unequal variances, we found statistically significant higher values in boys with autism and control boys: *p =* 0.028 (Tamhane’s test) (Figure 4). Also, in general, girls from both groups had higher serine, threonine, and aspartic acid values than boys from both groups but without statistical significance (Figure 4).

When comparing the mean values for levels of urine amino acids by sex and age for the autism group, we found correlations between boys under 5 years old and boys over 5 years old for aspartic acid (*p* = 0.001), glutamic acid (*p* = 0.009), alanine (*p* = 0.003), glutamine (*p* = 0.027), histidine (*p* = 0.008), tyrosine (*p* = 0.038), and valine (*p* = 0.001). When comparing the mean values for girls and age under 5 years old and over 5 years old, we found no statistical correlations (Table 3).

### 3.3. Analysis of Amino Acids in Urine According to Age for the Study Group and the Control Group

After performing the general analysis according to the age groups, we found statistically significant values between the study group and the control group: DIF = 314.077, *p =* 0.018. We found higher, statistically significant values for histidine, threonine, valine, methionine, aspartic acid, glutamic acid, alpha amino-adipic acid, sarcosine, alanine, and beta-alanine and significantly lower values for proline in relation to the age group under 5 years for the two groups (Table 4).

**Figure 5:**
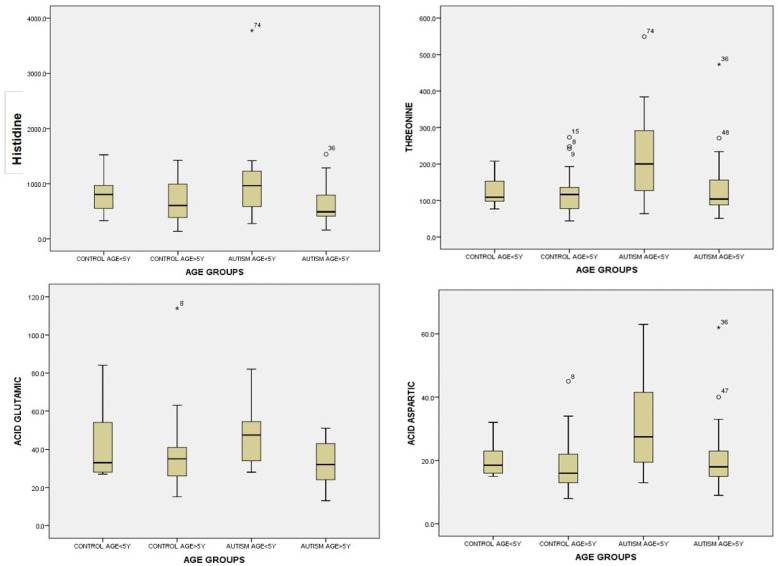
Urine histidine, threonine, glutamic acid, and aspartic acid levels by age < 5 years and >5 years for the control group and study group (autism). Cases with extreme values outside the 95% CI (outliers) are represented by circles and asterisks on the boxplot.

For urinary histidine, ANOVA was significant across groups: F(3) = 2.749; *p* = 0.049 (Table S4). Children with autism aged < 5 years had much higher values of histidine in urine, compared to children in the same group aged > 5 years and compared to children in the control group regardless of age (Table S5, Figure 5).

For urinary threonine, after applying ANOVA across groups, we found statistical significance between the group of children with autism and the control group: F(3) = 4.194, *p* = 0.009. After analyzing threonine values with Bonferroni correction, we observed statistically significant differences in urinary threonine. Children with autism < 5 years had higher urinary threonine values compared to children with autism > 5 years: 213.85 ± 121.76 versus 136.44 ± 89.49, *p* = 0.033, as well as compared to the control group of children under 5 years, 213.85 ± 121.76 versus 125.62 ± 46.28 (Table S6, Figure 5).

For aspartic acid in urine, we found statistically significant values in the ANOVA analysis over groups, *p =* 0.006 (Table S7). For multiple comparisons, we used the Bonferroni method and found statistically significant results by age group. Children with autism < 5 years had higher aspartic acid values: 30.40 ± 13.87 compared to children with autism > 5 years: 21.12 ± 11.49, *p =* 0.041 and compared to children in the group of study > 5 years: 18.63 ± 8.96, *p =* 0.006, but without significance between groups under 5 years of age in the two groups (Table S8, Figure 5).

For glutamic acid in urine, we found statistically significant values in the ANOVA analysis, *p =* 0.040 (Table S9). After performing Bonferroni correction for multiple comparisons between groups, we found that there was statistical significance within the study group. Children with autism < 5 years had higher urinary concentrations of glutamic acid compared to children with autism aged > 5 years: dif = 13.88, *p =* 0.033 (Table S10, Figure 5).

**Figure 6:**
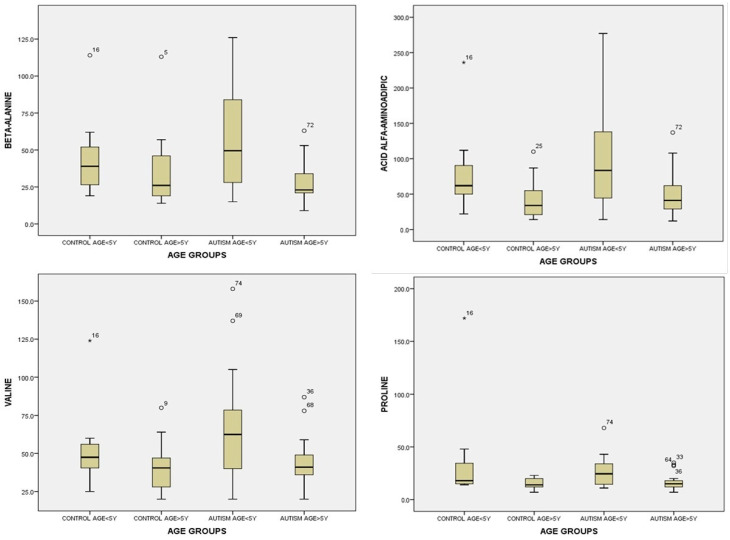
Urine beta-alanine, alpha aminoadipic acid, valine, and proline levels by age: <5 years and >5 years for the control group and study group (autism). Circles and asterisks represent the cases with extreme values outside the 95% CI (outliers).

For beta-alanine in urine, we applied ANOVA across groups and found statistically significant values by age group between the group of children with autism and the control group, F(3) = 6.066, *p =* 0.001 (Table S11). Regarding urinary beta-alanine, for multiple comparisons, using the Tamhane test for unequal variances, we obtained statistically significant values between the group of children with autism < 5 years: 58.95 ± 36.18 and the group of children with autism > 5 years: 28.32 ± 13.54, *p =* 0.009. Children with autism < 5 years old had higher values of urinary beta-alanine compared to children with autism > 5 years old (Table S12, Figure 6).

For alpha amino-adipic acid in urine, after performing the statistical analysis using ANOVA, we found statistically significant differences between the studied groups, *p =* 0.001 (Table S13). For multiple comparisons between groups of alpha amino-adipic acid values in urine, we found significant differences between the group of children with autism < 5 years: 99.80 ± 70.80 and the group children with autism > 5 years: 46.60 ± 29.99, *p =* 0.026, as well as the control group > 5 years: 43.18 ± 27.33, *p =* 0.016. Children with autism < 5 years old had higher alpha amino-adipic acid values than children from both groups > 5 years old (Table S14, Figure 6).

For valine in urine, in the ANOVA test, we found statistically significant results: F(3) = 5.491, *p =* 0.002. For multiple comparisons between groups of valine values, we used Tamhane’s test for unequal variances. We found significant differences between the group of children with autism < 5 years: 67.75 ± 34.73 and the group of children with autism > 5 years: 43.92 ± 15.36, *p =* 0.050, as well as the control group > 5 years: 40.95 ± 14.96, *p = 0.022.* Children with autism < 5 years old had significantly higher values compared to children > 5 years old in both groups, but they were statistically insignificant compared to the group under 5 years old in the control group (Table S15, Figure 6).

For multiple comparisons between groups of urinary proline values, we used Tamhane’s test for unequal variances. We found significant differences between the group of children with autism < 5 years: 6.85 ± 14.10 and the group of children with autism > 5 years: 16.36 ± 7.26, *p =* 0.032, as well as the control group > 5 years: 15.13 ± 5.03, *p =* 0.011. Children with autism < 5 years old had significantly lower values compared to children under 5 years old from the control group (Table S16, Figure 6).

**Figure 7:**
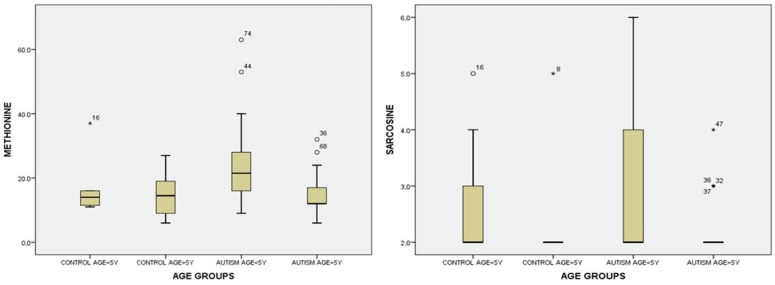
Urine methionine and sarcosine levels by age: <5 years and >5 years for the control group and study group (autism). Cases with extreme values outside the 95% CI (outliers) are represented.

For methionine in urine, we found statistically significant values between the group of children with autism < 5 years and the group of children with autism > 5 years, *p =* 0.004 (Table S17). Children with autism < 5 years old had significantly higher values compared to children with autism > 5 years old (Figure 7).

For the analysis of sarcosine in urine by age groups, since the criteria for the Gaussian distribution were not met, we used the non-parametric Kruskal–Wallis test and found statistically significant differences over the groups, *p =* 0.037, but we found no statistical significance between the under-five groups in the two groups (Table S18). When we used Mann–Whitney tests, we found significance only between the control group > 5 years and the autistic group < 5 years, *p* = 0.007. These were considered cross meanings and were not taken into account (Table S19, Figure 7).

For the analysis of urinary alanine, we used Mann–Whitney tests and found significance between the control group > 5 years and the autistic group < 5 years, *p =* 0.018. These findings were considered cross meanings, and they were not taken into account (Table S20).

## 4. Discussion

The amino acids, the building blocks of peptides and proteins, play crucial roles in maintaining various bodily functions. The absorption of dietary amino acids is facilitated by numerous amino acid transporters, with some amino acids being derived directly from food while others are synthesized endogenously. These molecules serve as precursors for a wide array of proteins, contributing efficiently to bodily processes. Amino acid metabolism is closely regulated by the gastrointestinal and urinary systems, particularly the kidneys, which play vital roles in this process [10]. Notably, urine analysis can provide valuable insights into metabolic disorders or nutritional imbalances, given its composition of numerous metabolites [11]. In children with autism spectrum disorder (ASD), urinary metabolites may reflect dysfunctions across various pathways implicated in the disorder’s etiology, including oxidative stress, inflammation, mitochondrial dysfunction, and intestinal microbiome dysregulation [12].

Upon conducting statistical analysis, significant differences in urinary amino acid profiles were observed between children with ASD and those without psychiatric pathology. Specifically, the overall descriptive analysis revealed differences in glutamine, serine, hydroxyproline, tyrosine, aspartic acid, and tryptophan levels between the two groups. Furthermore, distinctions were noted based on sex, with variations in serine, threonine, and aspartic acid levels, and on age, with differences in histidine, threonine, proline, valine, methionine, aspartic acid, glutamic acid, alpha-amino adipic acid, sarcosine, and beta-alanine levels. Interestingly, significant differences were observed in certain amino acids among autistic boys based on age, including aspartic acid, glutamic acid, alanine, glutamine, histidine, tyrosine, and valine. However, such significance was not observed among girls or with respect to age. Nevertheless, the small sample size may have influenced these findings, necessitating further investigation to elucidate age and sex-related differences. Although various discriminating metabolites were identified in plasma and urine samples, metabolic pathway analysis indicated disruptions in pathways associated with taurine metabolism, phenylalanine metabolism, and arginine and proline metabolism, evident in both plasma and urine [13].

Statistically significant differences in urinary levels of aspartic acid were observed between children with autism and those without psychiatric pathology (*p* = 0.029). Children with autism exhibited higher levels of aspartic acid compared to their counterparts, with autistic boys showing particularly elevated values. Furthermore, age stratification revealed that children with autism under 5 years old had higher levels of aspartic acid compared to older autistic children and controls. Aspartic acid, an endogenous amino acid, serves various neuroendocrine functions and acts as a precursor to neurotransmitters like N-methyl-d-aspartic acid (NMDA), crucial for nervous system development and function [14]. Moreover, asparagine synthetase, which converts aspartate to asparagine, is essential for neurological health, and its deficiency can lead to neurological symptoms such as seizures and intellectual disabilities [15].

Similarly, glutamine levels showed significant differences (*p* = 0.032) between children with autism and controls, with the former exhibiting higher levels. Glutamine, a non-essential amino acid, plays vital roles in metabolism, cellular integrity, protein synthesis, and redox potential regulation. It can also influence metabolic pathways and cellular defense mechanisms, impacting overall body homeostasis [16]. Interestingly, low urinary glutamine levels have been associated with improved autism spectrum symptoms [17]. These findings suggest a potential link between glutamine metabolism and ASD symptoms, warranting further investigation.

Serine, another essential amino acid, was significantly lower in children with autism compared to controls. Serine plays crucial roles in protein synthesis, neurotransmission, and folate and methionine cycles, and its deficiency can lead to nervous system dysfunctions [18]. External supplementation with serine compounds has shown promise in alleviating various neurological conditions, including psychosis, schizophrenia, and epilepsy [19]. In this study, autistic boys exhibited significantly lower serine levels compared to control boys, while girls generally had higher serine levels regardless of their group, though not statistically significant. These findings suggest a potential association between low serine levels and ASD symptoms, particularly in boys. However, further studies with larger sample sizes are needed to validate these hypotheses.

For hydroxyproline, the Mann–Whitney test for non-parametric distributions was used, and we obtained statistically significant differences between the values of the two groups: U = 493.5, *p* = 0.048. Children with autism had significantly higher values compared to children in the control group.

Tyrosine is an amino acid that appears in the human organism during embryogenesis, being catalyzed by the enzyme protein kinase, with an important role in cell signaling pathways [20]. Tyrosine is involved in cell signaling, cell proliferation, homeostasis of metabolism, activation of cellular transcription, neuronal transmission, differentiation, development, and aging. Thus, the dysfunction of tyrosine or its enzyme has been associated with many diseases including cancer [21]. Tyrosine and phenylalanine are involved in the synthesis of dopamine, which seems to be involved in the etiological mechanism of autism. Tyrosine is also involved in the proper functioning of the intestinal microbiome [22]. A study by Timperio et al. in 2022 hypothesized that the low levels of tyrosine and DOPA in the urine of children with autism are due to a deficiency of the enzyme phenylalanine hydroxylase, which leads to a destabilization of the tyrosine–phenylalanine balance, with the imbalance of the gut microbiome and aggravation of autism spectrum symptoms [23]. This hypothesis, issued by Timperio et al., is not proven in terms of the groups of children studied in the present research. We found that tyrosine values for children with autism were higher than children without psychiatric pathology, *p* = 0.043. It is possible that this change in tyrosine values alters the gut microbiome and amino acid metabolism, but the mechanism remains to be investigated.

Tryptophan, known for its association with ASD symptoms, acts as a precursor to serotonin (5-HT), an inhibitory monoamine neurotransmitter recognized as a biomarker in ASD [24]. However, current research on urinary tryptophan levels in ASD remains inconclusive, with some studies reporting increased values while others find low levels [25]. Tryptophan plays a crucial role not only in serotonin metabolism but also in melatonin synthesis, further implicating its significance in ASD [24]. Low tryptophan levels have been linked to autism spectrum symptoms [24]. In a study by Xu XJ et al., neurotransmitter-associated metabolites exhibited a significantly higher number of correlates in the control group compared to the ASD group, with no significant correlation observed between serotonin and its precursor tryptophan in the ASD cohort [15,22]. Surprisingly, our study found that children with autism had significantly higher urinary tryptophan levels compared to children in the control group, contrary to existing literature [25]. Bent et al. demonstrated negative correlations between tryptophan and tyrosine levels in urine and autism spectrum symptoms, suggesting that elevated levels of these amino acids may exacerbate symptoms [17]. Taking Bent’s hypothesis into account, the increased levels of tyrosine and tryptophan observed in our study may contribute to the severity of autistic-type symptoms [17].

For threonine, boys in the autism group exhibited significantly higher values compared to boys in the control group. Threonine, belonging to the aspartate family, serves as a proteinogenic amino acid [24]. Its breakdown generates acetylcholinesterase A and glycine, contributing to various physiological processes and overall body homeostasis [25]. Pairwise analysis revealed statistically significant differences in urinary threonine, with children under 5 years of age with autism exhibiting higher levels than those over 5 years old: 213.85 ± 121.76 versus 136.44 ± 89.49, *p* = 0.033.

Regarding proline, children under 5 years old with autism had notably lower values compared to those over 5 years old in both groups. Hidding E. et al. demonstrated that abnormally elevated proline levels, associated with the CMPT158 genotype of chromosome 22q11.2 deletion, impact the severity of autism spectrum symptoms, particularly affecting facial emotion recognition, behavior, and cognition [26].

In the case of valine, children under 5 years old with autism displayed significantly higher values compared to their older counterparts in both groups. Valine, an essential amino acid requiring external dietary sources, serves as a precursor to methylmalonyl coenzyme A, essential for vitamin B12 synthesis and urinary excretion in excess [27]. Similarly, histidine metabolism necessitates the removal of the formimic group from glutamic acid [28].

Concerning methionine, children under 5 years old with autism exhibited markedly higher values than those over 5 years old. Current research suggests that abnormal methylation increases the risk of autism spectrum symptoms [17], underscoring the pivotal role of the methionine cycle in the methylation process. Methionine serves as a crucial precursor to cystine and cysteine [29].

Children under 5 years old with autism exhibited higher alpha amino-adipic acid values compared to children in both groups over 5 years old. The alpha amino adipic acid pathway contributes to lysine synthesis [30]. Analyzing sarcosine by age group using the non-parametric Kruskal–Wallis test due to non-Gaussian distribution, we found statistically significant differences across groups. Sarcosine, a derivative of glycine and an intermediate metabolite, belongs to the N-methyl amino acid type [31]

Histidine and beta-alanine exert antioxidant effects by scavenging oxygen radicals, thereby participating in oxidative stress mitigation. Chen et al.’s 2019 study revealed significantly elevated markers of oxidative stress in urine samples of children with autism [32]. For histidine, ANOVA showed significance across groups (*p* = 0.049). Children under 5 years old with autism displayed notably higher values compared to both older autistic children and the control group, irrespective of age. Moreover, urinary beta-alanine values were higher in children under 5 years old with autism compared to those over 5 years old. Saleem’s 2018 study indicated decreased levels of aspartic acid, glycine, beta-alanine, tryptophan, lysine, and proline and increased asparagine values in the serum of children with autism compared to controls [33]. However, this study focused on serum amino acids, and extrapolating these findings to urine amino acid profiles requires caution. Our urine amino acid profile analysis revealed increased urinary values in the autistic group for aspartic acid, glutamine, asparagine, hydroxyproline, tryptophan, tyrosine, threonine, valine, and methionine and decreased values for serine and proline.

Human urine serves as a valuable resource for biochemical screening, offering insights into various physiological conditions and nutritional statuses. Alterations in urinary metabolite levels may stem from diverse factors such as metabolic anomalies, medication use, dietary patterns, or other physiological states [34,35].

## 5. Limitations of the Study

Limited sample size: The sample size in our study was relatively small, with 45 children diagnosed with autism spectrum disorder (ASD) and 30 control children. A larger sample size would enhance the statistical power and generalizability of the findings.

Potential confounding factors: We did not adequately control for potential confounding factors such as diet, medication use, comorbidities, and autism severity. These factors could influence urinary amino acid levels and may have affected the study results.

Cross-sectional design: The cross-sectional design of our study limits our ability to establish causality or temporal relationships between urinary amino acid levels and autism spectrum disorder (ASD). Longitudinal studies would provide more insights into the dynamic changes in amino acid metabolism over time.

Measurement technique: While liquid chromatography–tandem mass spectrometry (LC-MS/MS) is a robust technique for amino acid analysis, variations in sample collection, storage, and processing could introduce measurement error. Future studies should ensure standardized protocols to minimize these potential sources of bias.

Generalizability: Our study focused on a specific cohort of Romanian children aged between 2 and 12 years, limiting the generalizability of the findings to other populations. Replication of the study in diverse populations would strengthen the external validity of the results.

## 6. Conclusions

Based on the comprehensive analysis provided, the study offers compelling insights into the intricate relationship between urinary amino acid profiles and autism spectrum disorder (ASD). By identifying significant differences in amino acid levels between children with ASD and those without psychiatric pathology, the research underscores the potential dysfunctions within various metabolic pathways associated with ASD, including oxidative stress, inflammation, mitochondrial dysfunction, and intestinal microbiome dysfunctions.

Moreover, the study’s nuanced exploration of sex and age disparities in urinary amino acid levels highlights the complexity of ASD pathology. Notably, the higher levels of specific amino acids in autistic boys and younger children with ASD illuminate potential avenues for targeted interventions. For instance, the observed deficiencies in amino acids like serine in children with ASD suggest a plausible therapeutic target for addressing cognitive symptoms associated with the disorder.

Furthermore, the study’s findings regarding the roles of amino acids such as tyrosine and tryptophan in neurotransmitter synthesis and gut microbiome function offer promising avenues for future research and therapeutic development. Understanding how these amino acids influence neurodevelopmental processes could pave the way for innovative treatment strategies aimed at mitigating ASD symptoms and improving overall quality of life for affected individuals.

In conclusion, this study underscores the significance of urinary amino acid analysis as a valuable tool for elucidating the metabolic dysregulation underlying ASD. By shedding light on the intricate interplay between amino acid metabolism and ASD pathology, the research opens new avenues for targeted interventions and personalized treatment approaches tailored to the specific metabolic profiles of individuals with ASD.

## Figures and Tables

**Table 1:** Distribution of amino acids in urine as a function of mean concentration expressed in μmol/L and standard deviation for the study group (autism) and the control group.

Urine Amino Acids	Autism Group (*n* = 45) Mean ± SD	Control Group (*n* = 30) Mean ± SD	*p*-Value
Alpha amino butyric acid	18.44 ± 14.21	17.53 ± 14.01	0.787
Arginine	16.08 ± 12.52	14.93 ± 8.06	0.638
Aspartic acid	25.24 ± 13.30	19.06 ± 8.16	0.029
Asparagine	69.93 ± 63.30	51.03 ± 33.32	0.185
Citrulline	4.60 ± 3.38	3.53 ± 2.89	0.104
Cystine	16.60 ± 9.35	14.33 ± 5.91	0.223
Glutamic acid	38.28 ±14.04	39.16 ± 20.44	0.826
Glutamine	678.60 ± 362.71	511.23 ± 295.45	0.032
Glycine	1189.51 ± 710.21	1159.60 ±1401.79	0.114
Histidine	811.11 ± 580.99	706.00 ± 354.46	0.334
Hydroxyproline	13.28 ± 22.16	7.40 ± 2.87	0.048
Isoleucine	32.28 ± 17.15	28.00 ± 14.51	0.14
Leucine	54.60 ± 30.54	46.33 ± 18.80	0.190
Lysine	74.04 ± 83.39	69.03 ± 50.60	0.747
Methionine	18.86 ±11.42	15.03 ± 6.56	0.162
Methylhistidine	248.46 ± 144.30	235.40 ± 217.19	0.773
Ornithine	18.64 ± 25.01	20.93 ± 16.75	−0.502
Phenylalanine	98.62 ± 57.86	85.36 ± 42.64	0.216
Proline	21.02 ± 11.93	21.80 ± 29.32	0.283
Serine	259.00 ± 105.83	327.93 ± 188.04	0.047
Taurine	617.31 ± 650.83	432.96 ± 356.31	−0.361
Threonine	170.84 ± 110.83	126.26 ± 59.77	0.143
Tryptophan	95.93 ± 47.69	75.76 ± 29.95	0.028
Tyrosine	241.46 ± 113.05	198.80 ± 66.25	0.043
Valine	54.51 ± 28.16	44.60 ± 20.38	0.119

*p*: ANOVA test results for multiple comparisons, with a significant *p* < 0.05; t: Student’s *t*-test with a significant *p*-value < 0.05.

**Table 2:** Mean of urinary amino acid concentrations expressed in μmol/L and standard deviation by sex for the autism group and the control group.

Urine Amino Acids	Autism Group (*n*= 45) Mean ± SD		Control Group (*n* = 30) Mean ± SD	
	Boys (*n* = 35)	Girls (*n* = 10)	Boys (*n* = 13)	Girls (*n* = 17)
Alpha aminoadipic acid	71.14 ± 62.24	67.10 ± 42.71	42.07 ± 20.27	62.70 ±54.89
Alpha aminobutyric acid	16.94 ± 11.38	23.70 ± 21.42	13.00 ± 6.11	21.00 ±17.27
Alanine	354.65 ± 256.45	468.40 ± 219.06	252.84 ± 187.13	338.64 ± 235.85
Arginine	17.00 ± 13.89	12.90 ± 4.81	15.15 ± 8.17	14.76 ± 8.22
Asparagine	70.37 ± 67.18	68.40 ± 50.32	40.00 ± 24.00	59.47 ± 37.50
Aspartic acid	25.05 ± 13.21	25.90 ± 14.29	16.76 ± 5.97	20.82 ± 9.29
Betha-alanine	40.57 ± 30.62	46.70 ± 28.91	37.46 ± 25.83	36.17 ± 24.82
Citrulline	4.68 ± 3.61	4.30 ± 2.58	2.76 ± 1.48	4.11 ± 3.56
Cystine	16.28 ±10.36	17.70 ± 4.52	13.46 ± 5.23	15.17 ± 6.43
Glutamic acid	36.20 ± 13.34	45.60 ± 14.69	34.84 ± 12.16	42.47 ± 24.90
Glutamine	660.28 ± 380.50	742.70 ± 300.59	408.61 ± 194.19	589.70 ± 338.87
Glycine	1098.80 ± 662.04	1507.00 ± 816.18	922.07 ± 1042.37	1341.23 ± 1632.73
Histidine	820.88 ± 625.24	776.90 ± 414.40	621.15 ± 230.85	770.88 ± 421.23
Hydroxyproline	9.94 ± 6.48	25.00 ± 45.23	6.92 ± 2.69	7.76 ± 3.03
Isoleucine	32.31 ± 16.50	32.20 ± 20.26	25.61 ± 8.24	29.82 ± 17.96
Leucine	54.77 ± 30.15	54.00 ± 33.54	42.84 ± 11.42	49.00 ± 22.92
Lysine	253.31 ± 150.77	231.50 ± 124.56	235.53 ± 193.25	235.29 ± 239.76
Methionine	18.74 ± 11.91	19.30 ± 10.04	12.61 ± 3.64	16.88 ± 7.72
Methylhistidine	253.31 ± 150.77	231.50 ± 124.56	235.53 ± 193.25	235.29 ± 239.76
Ornithine	18.45 ± 27.00	19.30 ± 17.42	20.53 ± 16.15	21.23 ± 17.69
Phenylalanine	97.88 ± 55.42	101.20 ± 68.95	79.61 ± 31.93	89.76 ± 49.83
Proline	21.00 ± 12.42	21.10 ± 10.66	16.46 ± 10.16	25.88 ± 37.95
Sarcosine	2.62 ±1.21	2.50 ± 0.97	2.00 ± 0.00	2.47 ± 1.06
Serine	322.82 ± 170.75	345.80 ± 249.52	215.61 ± 77.86	292.17 ±114.26
Taurine	558.91 ± 618.59	821.70 ± 752.03	364.00 ± 303.36	485.70 ± 392.77
Threonine	170.40 ± 109.42	172.40 ± 121.721	100.69 ± 38.02	145.82 ±66.73
Tyrosine	243.37 ± 113.68	234.80 ± 116.62	184.76 ± 51.76	209.52 ± 75.26
Tryptophan	98.77 ± 51.069	86.00 ± 33.546	74.15 ± 26.82	77.00 ± 32.91
Valine	54.42 ± 26.84	54.80 ± 33.96	39.46 ±11.68	48.52 ± 24.76

n = number of participants, SD = standard deviation.

**Table 3:** Mean of urinary amino acid concentrations expressed in μmol/L and standard deviation by sex and age for the autism group.

Urine Amino Acids	Autism Group (*n* = 45) Mean ± Standard Deviation			
	Boys < 5 Years (*n* = 15)	Boys ≥ 5 Years (*n* = 20)	Girls < 5 Years (*n* = 5)	Girls ≥ 5 Years (*n* = 5)
Alpha aminoadipic acid	105.00 ± 76.94	45.75 ± 31.44	84.20 ± 51.85	50.00 ± 26.20
Alpha aminobutiric acid	22.40 ± 13.80	12.85 ± 7.10	31.00 ± 28.47	16.40 ± 9.45
Aspartic acid	32.40 ± 14.94	19.55 ± 8.59	24.40 ± 8.53	27.40 ± 19.53
Glutamic acid	44.73 ± 11.16	29.80 ± 11.24	49.80 ± 20.40	41.40 ± 5.03
Alanine	491.67 ± 315.09	251.90 ± 136.41	440.60 ± 224.85	496.20 ± 235.55
Anserine	3.13 ± 3.44	1.70 ± 1.08	4.40 ± 3.78	1.00 ± 0.00
Arginine	18.13 ± 6.15	16.15 ± 17.77	12.60 ± 2.51	13.20 ± 6.76
Asparagine	96.20 ± 92.42	51.00 ± 29.38	73.00 ± 57.33	63.80 ± 48.58
Beta-alanine	59.93 ± 36.71	26.05 ± 12.94	56.00 ± 38.58	37.40 ± 13.28
Citrulline	5.47 ± 3.52	4.10 ± 3.65	5.40 ± 3.05	3.20 ± 1.64
Cystathionine	9.73 ± 6.05	6.10 ± 4.60	7.80 ± 6.57	10.60 ± 7.09
Cystine	17.07 ± 6.97	15.70 ± 12.47	18.00 ± 3.67	17.40 ± 5.68
Phenilalanine	121.27 ± 73.60	80.35 ± 27.40	116.40 ± 96.43	86.00 ± 28.64
Glutamine	807.60 ± 480.75	549.80 ± 242.50	772.60 ± 346.52	712.80 ± 284.59
Glycine	1383.87 ± 741.77	885.00 ± 516.37	1381.00 ± 636.50	1633.00 ± 1026.66
Histidine	1085.33 ± 822.29	622.55 ± 323.62	837.60 ± 395.22	716.20 ± 470.09
Homocystine	2.00 ± 0.00	2.00 ± 0.00	2.00 ± 0.00	2.00 ± 0.00
Hydroxylysine	13.27 ± 8.00	9.95 ± 5.78	15.20 ± 13.10	12.20 ± 3.56
Hydroxyproline	13.87 ± 7.91	7.00 ± 2.81	38.60 ± 64.08	11.40 ± 5.94
Isoleucine	41.73 ± 19.62	25.25 ± 9.00	33.40 ± 29.27	31.00 ± 8.00
Leucine	70.93 ± 35.28	42.65 ± 18.69	56.20 ± 41.21	51.80 ± 28.67
Lysine	81.67 ± 58.61	72.95 ± 108.51	94.00 ± 76.21	35.60 ± 12.86
Methionine	25.20 ± 15.16	13.90 ± 5.21	21.80 ± 11.12	16.80 ± 9.36
Methylhistidine	283.47 ± 192.33	230.70 ± 110.31	220.00 ± 122.00	243.00 ± 140.36
Ornithine	25.13 ± 40.17	13.45 ± 7.37	22.80 ± 19.85	15.80 ± 16.07
Proline	28.60 ± 14.82	15.30 ± 5.89	21.60 ± 11.44	20.60 ± 11.15
Sarcosine	3.20 ± 1.61	2.20 ± 0.52	2.60 ± 1.34	2.40 ± 0.55
Serine	410.73 ± 205.79	256.90 ± 101.47	268.00 ± 181.96	423.60 ± 303.06
Taurine	648.33 ± 800.05	491.85 ± 449.53	1024.40 ± 1036.08	619.00 ± 310.37
Threonine	235.00 ± 126.92	121.95 ± 61.59	150.40 ± 85.65	194.40 ± 157.45
Tryptophan	117.80 ± 66.62	84.50 ± 29.94	92.80 ± 43.17	79.20 ± 23.51
Tyrosine	283.67 ± 130.01	213.15 ± 91.80	246.20 ± 126.14	223.40 ± 119.87

**Table 4:** Descriptive analysis of urinary amino acids by age for the study group and the control group.

Urine Amino Acids	Autism Group (*n*= 45) Mean ± SD		Control Group (*n* = 30) Mean ± SD		*p*-Value
	Age < 5 Years (*n* = 25)	Age > 5 Years (*n* = 20)	Age < 5 Years (*n* = 15)	Age > 5 Years (*n* = 15)	
Alanine	478.90 ± 290.36	300.76 ± 184.18	296.87 ± 154.24	303.13 ± 238.81	0.018
Alpha amino adipic acid	99.80 ± 70.80	46.60 ± 29.99	82.87 ± 66.91	43.18 ± 27.33	0.010
Alpha aminobutyric acid	24.55 ± 18.04	13.56 ± 7.54	23.37 ± 23.96	15.40 ± 7.87	*0.787*
Arginine	16.75 ± 5.93	15.56 ± 16.09	19.00 ± 12.44	13.45 ± 5.44	*0.613*
Asparagine	90.40 ± 84.21	53.56 ± 33.22	51.75 ± 30.95	50.77 ± 34.84	*0.213*
Aspartic acid	30.40 ± 13.87	21.12 ± 11.49	20.25 ± 5.72	18.63 ± 8.96	0.006
Betha-alanine	58.95 ± 36.18	28.32 ± 13.545	46.00 ± 30.63	33.36 ± 22.22	0.001
Lysine	267.60 ± 176.59	132.40 ± 113.76	233.16 ± 383.61	187.40 ± 83.90	0.080
Cystine	17.30 ± 6.23	16.04 ± 11.35	15.75 ± 8.54	13.95 ± 4.79	*0.621*
Citrulline	5.45 ± 3.33	3.92 ± 3.34	3.75 ± 1.03	3.45 ± 3.34	*0.211*
Serine	375.05 ± 205.41	290.24 ± 167.60	244.00 ± 57.65	264.45 ± 119.34	*0.094*
Glycine	1383.15 ± 700.51	1034.60 ± 692.82	901.37 ± 445.11	1253.50 ± 1616.46	*0.590*
Glutamic acid	46.00 ±13.58	32.12 ± 11.25	42.62 ± 20.30	37.90 ± 20.82	0.040
Glutamine	798.850 ± 442.51	582.40 ± 253.93	584.00 ± 246.92	484.77 ± 312.20	0.025
Histidine	1023.40 ± 737.03	641.28 ± 348.14	812.75 ±365.17	667.18 ± 350.91	0.049
Hydroxyproline	20.050 ± 32.115	7.880 ± 3.919	7.875 ± 3.090	7.22 ±3.91	*0.055*
Isoleucine	39.65 ± 21.85	26.40 ± 8.96	36.75 ± 23.29	24.81 ± 8.38	*0.050*
Leucine	67.25 ± 36.29	44.48 ± 20.67	48.62 ± 24.48	45.50 ± 16.90	*0.106*
Methionine	24.35 ± 14.06	14.48 ± 6.12	16.37 ± 8.56	14.54 ± 5.83	0.005
Metylhistidine	2.32 ±1.37	2.00 ±1.12	1.80 ±0.67	2.46 ±0.91	0.025
Ornithine	24.55 ± 35.67	13.92 ± 9.32	26.00 ± 20.80	19.09 ± 15.17	*0.890*
Phenylalanine	120.05 ± 77.16	81.48 ± 27.13	84.75 ± 29.74	85.59 ± 47.07	*0.063*
Proline	26.85 ± 14.10	16.36 ± 7.26	40.12 ± 54.44	15.13 ± 5.03	0.032
Sarcosine	3.05 ±1.53	2.24 ±0.52	2.62 ±1.18	2.13 ±0.63	0.007
Taurine	742.35 ± 851.78	517.28 ± 422.76	419.50 ± 364.37	437.86 ± 361.90	0.019
Threonine	213.85 ± 121.76	136.44 ±89.49	125.62 ±46.28	126.50 ±64.96	0.009
Tyrosine	274.30 ± 126.81	215.20 ±95.31	210.00 ±61.97	194.72± 68.68	*0.055*
Tryptofan	111.55 ± 61.53	83.44 ± 28.39	80.87 ± 24.47	73.90 ± 32.03	0.006
Valine	67.75 ± 34.73	43.92 ± 15.36	54.62 ± 29.89	40.95 ± 14.96	0.002

SD = standard deviation, n = number of subjects, *p* = ANOVA test result for multiple comparisons with significance threshold *p* < 0.05, f(3) = ANOVA ratio.

## Data Availability

We confirm that the main data supporting the findings of this study are available within the article, and any other additional data are available on request.

## Supplementary Materials

The following supporting information can be downloaded at: https://www.mdpi.com/article/10.3390/life14050629/s1, Table S1. Comparison of urinary amino acid means using nonparametric tests (Mann-Whitney test and Wilcoxon test); Table S2. Urinary aminoacids. ANOVA test; Table S3. Comparative analysis of urinary serine. threonine and aspartic acid values for the study group (autism) and the control group according to male (m) and female (f) gender using the Tamhane Test and the Dunnet Test; Table S4. ANOVA analysis across groups and within groups (autism and control) for urinary histidine values; Table S5. Tukey and Bonferroni analysis for multiple comparisons by age group for urinary histidine; Table S6. Bonferroni analysis for multiple comparisons by age group for urinary threonine; Table S7. ANOVA across groups for between-group comparisons for urinary aspartic acid; Table S8. Bonferroni analysis for multiple comparisons by age for urinary aspartic acid; Table S9. Between-group ANOVA analysis for urinary glutamic acid values; Table S10. Bonferroni correction for multiple comparisons by age between groups for urinary glutamic acid; Table S11. ANOVA test for the comparative analysis of urinary beta-alanine by age between the control group and the control group; Table S12. Tamhane test for multiple comparisons between urinary beta-alanine values according to age groups for the studied groups (autism and control); Table S13. ANOVA test for comparison of urinary alpha-amino-adipic acid by age between controls and controls; Table S14. Tamhane test for multiple comparisons by age group for urinary alpha aminoadipic acid; Table S15. Tamhane multiple comparisons test by age for urinary valine; Table S16. Tamhane test for multiple comparisons. according to age groups for proline in urine; Table S17. Age group Mann–Whitney for urinary methionine; Table S18. Descriptive analysis for urinary sarcosine by age and Kruskal Wallis Test for multiple comparisons between groups; Table S19. Mann–Whitney test for sarcosine in urine; Table S20. Mann–Whitney Test for urinary alanine.
