# Supplementary material for: A Clinical Study of Urine Amino Acids in Children with Autism Spectrum Disorder
Source: Life (Basel). 2024 May 15;14(5):629. doi: 10.3390/life14050629 (PMC11123416; doi:10.3390/life14050629)
Supplement: Supplementary file 1 [file life-14-00629-s001.zip › life-2984897-supplementary.pdf]

Table S1. Comparison of urinary amino acid means using nonparametric tests (Mann-Whitney test and Wilcoxon test).

**Urine amino acids**	**U (Mann-Whitney)**	**W (Wilcoxon)**	**P**	**Z**
taurine	590.500	1055.500	0.361	-0.914
ornithine	613.000	1648.000	0.502	-0.672
asparagine	552.500	1017.500	0.185	-1.325
glicine	529.000	994.000	0.114	-1.579
citrulline	528.500	993.500	0.104	-1.627
hydroxyproline	493.500	958.500	0.048	-1.974
threonine	539.500	1004.500	0.143	-1.466
proline	576.000	1041.000	0.283	-1.073
alpha amino butiric acid	650.000	1115.000	0.787	-0.271
valine	531.000	996.000	0.119	-1.558
methionine	546.000	1011.000	0.162	-1.398
isoleucine	566.000	1031.000	0.238	-1.179
phenylalyearsne	560.500	1025.500	0.216	-1.239
aspartic acid	473.000	938.000	0.029	-2.189
sarcosine	554.500	1019.500	0.069	-1.820
alyearsne	535.500	1000.500	0.131	-0.131
Alpha amino-adipic acid	574.000	1039.000	0.275	-1.093
betha alyearsne	618.500	1083.500	0.541	-0.611

Table S2. Urinary aminoacids. ANOVA test

Urine amino acids	df	F	P
histidine	3	0.490	0.690
lisine	3	0.070	0.976
arginine	3	0.426	0.735
cystine	3	0.597	0.619
asparagine	3	$X^2(3) = 4.239$	0.237
glutamine	3	2.355	0.079
serine	3	1.715	0.172
glycine	3	0.805	0.495
citrulline	3	1.130	0.343
hydroxyproline	3	2.820	0.045
proline	3	0.515	0.673
Alpha amino butyric acid	3	$X^2(3)=8.720$	0.033
valine	3	1.213	0.311
methionine	3	1.385	0.254
tyrosine	3	1.309	0.278
isoleucine	3	0.577	0.632
leucine	3	0.702	0.554
phenylalyearsne	3	0.476	0.700
alpha amino adipic acid	3	0.958	0.417
betha alyearsne	3	0.327	0.806

F means Fisher's ratio = intergroup dispersion/intragroup dispersion (the higher the value of the ratio. the greater the dispersion of the sample means than the dispersion of the values of the null population) according to the DF (degree of freedom). ex : $F(3)=0.89$.

Table S3. Comparative analysis of urinary serine. threonine and aspartic acid values for the study group (autism) and the control group according to male (m) and female (f) gender using the Tamhane Test and the Dunnet Test

<table>
<tr><th rowspan="2">(I) Female (F)</th><th rowspan="2">(J) Male (M)</th><th rowspan="2">Mean differenc e (I-J)</th><th rowspan="2">Stand ard error</th><th rowspan="2">Sig.</th><th colspan="2">95% Confidence</th></tr>
<tr><th>Inferior limit</th><th>Superior limit</th></tr>
</table>

**Serine**						
Tamhane Test						
Control F	Control M	76.561	35.133	0.207	-22.950	176.073
	Autism F	-53.623	83.630	0.990	-319.631	212.384
	Autism M	-30.652	40.013	0.972	-140.788	79.484
Control M	Control F	-76.561	35.133	0.207	-176.073	22.950
	Autism F	-130.184	81.807	0.600	-395.101	134.732
	Autism M	-107.213*	36.047	0.028	-206.531	-7.896
Autism F	Control F	53.623	83.630	0.990	-212.384	319.631
	Control M	130.184	81.807	0.600	-134.732	395.101
	Autism M	22.971	84.018	1.000	-243.073	289.016
Autism M	Control F	30.652	40.013	0.972	-79.484	140.788
	Control M	107.213*	36.047	0.028	7.896	206.531
	Autism F	-22.971	84.018	1.000	-289.016	243.073
Dunnet Test						
Control F	Control M	76.561	35.133	0.198	-22.512	175.634
	Autism F	-53.623	83.630	0.984	-315.255	208.008
	Autism M	-30.652	40.013	0.968	-140.526	79.222
Control M	Control F	-76.561	35.133	0.198	-175.634	22.512
	Autism F	-130.184	81.807	0.543	-390.116	129.747
	Autism M	-107.213*	36.047	0.028	-206.288	-8.139
Autism F	Control F	53.623	83.630	0.984	-208.008	315.255
	Control M	130.184	81.807	0.543	-129.747	390.116
	Autism M	22.971	84.018	1.000	-238.849	284.792
Autism M	Control F	30.652	40.013	0.968	-79.222	140.526
	Control M	107.213*	36.047	0.028	8.139	206.288
	Autism F	-22.971	84.018	1.000	-284.792	238.849
**Threonine**						
Tamhane Test						
Control sex f	Control M	45.13	19.31	0.154	-9.832	100.094
	Autism F	-26.57	41.75	0.990	-157.239	104.086
	Autism M	-24.57	24.57	0.903	-92.068	42.915
Control M	Control F	-45.13	19.31	0.154	-100.094	9.832
	Autism F	-71.70	39.91	0.474	-200.940	57.525
	Autism M	-69.70*	21.29	0.012	-128.244	-11.171
Autism sex f	Control F	26.57	41.75	0.990	-104.086	157.239
	Control M	71.70	39.91	0.474	-57.525	200.940

	Autism M	2.00	42.70	1.000	-129.457	133.457
Autism	Control F	24.57	24.57	0.903	-42.915	92.068
sex m	Control M	69.70*	21.29	0.012	11.171	128.244
	Autism F	-2.00	42.70	1.000	-133.457	129.457
Acid aspartic în urină						
Tamhane Test						
Control F	Control M	4.054	2.797	0.645	-3.879	11.987
	Autism F	-5.076	5.051	0.912	-20.603	10.450
	Autism M	-4.233	3.174	0.716	-12.985	4.517
Control M	Control F	-4.054	2.797	0.645	-11.987	3.879
	Autism F	-9.130	4.814	0.407	-24.399	6.137
	Autism M	-8.287	2.781	0.028	-15.951	-0.625
Autism F	Control F	5.076	5.051	0.912	-10.450	20.603
	Control M	9.130	4.814	0.407	-6.137	24.399
	Autism M	0.842	5.042	1.000	-14.627	16.313
Autism M	Control F	4.233	3.174	0.716	-4.517	12.985
	Control M	8.287	2.781	0.028	0.625	15.951
	Autism F	-0.842	5.042	1.000	-16.313	14.627
Control F	Control M	4.054	2.797	0.622	-3.843	11.952
	Autism F	-5.076	5.051	0.886	-20.412	10.259
	Autism M	-4.233	3.174	0.702	-12.963	4.496
	Control F	-4.054	2.797	0.622	-11.952	3.843
	Autism F	-9.130	4.814	0.366	-24.153	5.892
Control M	Autism M	-8.287*	2.781	0.027	-15.932	-0.643
Autism F	Control F	5.076	5.051	0.886	-10.259	20.412
	Control M	9.130	4.814	0.366	-5.892	24.153
	Autism M	.842	5.042	1.000	-14.441	16.126
	Control F	4.233	3.174	0.702	-4.496	12.963
Autism M	Control M	8.287*	2.781	0.027	0.643	15.932
	Autism F	-0.842	5.042	1.000	-16.126	14.441

*. Mean difference is significant at $p < 0.05$

Table S4. ANOVA analysis across groups and within groups (autism and control) for urinary histidine values.

	Sum of squares	df	Mean of squares	F	Sig.
Between groups	1945582.054	3	648527.351	2.749	0.049
Within groups	16749484.613	71	235908.234		

Table S5. Tukey and Bonferroni analysis for multiple comparisons by age group for urinary histidine.

(I) age groups	(J) age groups	Mean difference (I-J)	Standard error	Sig.	95% Confidence	
					Inferior limit	Superior limit
			Tukey			
Control < 5 years	Control > 5 years	145.568	200.528	0.886	-382.009	673.146
	Autism < 5 years	-210.650	203.184	0.729	-745.215	323.915
	Autism > 5 years	171.470	197.293	0.821	-347.597	690.537
Control > 5 years	Control < 5 years	-145.568	200.528	0.886	-673.146	382.009
	Autism < 5 years	-356.218	150.061	0.092	-751.021	38.585
	Autism > 5 years	25.901	141.983	0.998	-347.649	399.452
Autism < 5 years	Control < 5 years	210.650	203.184	0.729	-323.915	745.215
	Control > 5 years	356.218	150.061	0.092	-38.585	751.021
	Autism > 5 years	382.120	145.711	0.051	-1.237	765.477
Autism > 5 years	Control < 5 years	-171.470	197.293	0.821	-690.537	347.597
	Control > 5 years	-25.901	141.983	0.998	-399.452	347.649
	Autism < 5 years	-382.120	145.711	0.051	-765.477	1.237
			Bonferroni			
Control < 5 years	Control > 5 years	145.568	200.528	1.000	-398.704	689.840
	Autism < 5 years	-210.650	203.184	1.000	-762.131	340.831
	Autism > 5 years	171.470	197.293	1.000	-364.023	706.963
Control > 5 years	Control < 5 years	-145.568	200.528	1.000	-689.840	398.704
	Autism < 5 years	-356.218	150.061	0.122	-763.514	51.078
	Autism > 5 years	25.901	141.983	1.000	-359.470	411.273
Autism < 5 years	Control < 5 years	210.650	203.184	1.000	-340.831	762.131
	Control > 5 years	356.218	150.061	0.122	-51.078	763.514
	Autism > 5 years	382.120	145.711	0.064	-13.368	777.608
Autism > 5 years	Control < 5 years	-171.470	197.293	1.000	-706.963	364.023
	Control > 5 years	-25.901	141.983	1.000	-411.273	359.470

	Autism < 5 years	-382.120	145.711	.064	-777.608	13.368

Table S6. Bonferroni analysis for multiple comparisons by age group for urinary threonine.

(I) age groups	(j) age groups	Mean difference (I-J)	Standard error	Sig.	95% Confidence Inferior limit	Superior limit
Control < 5 years	Control > 5 years	-0.875	37.236	1.000	-101.941	100.191
	Autism < 5years	-88.225	37.729	0.133	-190.630	14.180
	Autism > 5 years	-10.815	36.635	1.000	-110.251	88.621
Control > 5 years	Control < 5years	0.875	37.236	1.000	-100.191	101.941
	Autism < 5years	-87.350*	27.865	0.015	-162.981	-11.719
	Autism > 5 years	-9.940	26.365	1.000	-81.500	61.620
Autism < 5 years	Control < 5years	88.225	37.729	0.133	-14.180	190.630
	Control > 5 years	87.350*	27.865	0.015	11.719	162.981
	Autism > 5 years	77.410*	27.057	0.033	3.972	150.848
Autism > 5 years	Control < 5years	10.815	36.635	1.000	-88.621	110.251
	Control > 5 years	9.940	26.365	1.000	-61.620	81.500
	Autism < 5years	-77.410*	27.057	0.033	-150.848	-3.972

*. Mean difference is significant at $p < 0.05$

Table S7. ANOVA across groups for between-group comparisons for urinary aspartic acid

	Sum of squares	df	Mean of squares	F	Sig.
Between groups	1659.116	3	553.039	4.490	0.006
Within groups	8746.031	71	123.184		

Table S8. Bonferroni analysis for multiple comparisons by age for urinary aspartic acid

(I)age groups	(j) age groups	Mean difference (I-J)	Standard error	Sig.	95% Confidence Inferior limit	Superior limit
Control < 5 years	Control > 5 years	1.613	4.582	1.000	-10.824	14.051
	Autism < 5years	-10.150	4.643	0.193	-22.752	2.452
	Autism > 5 years	-0.870	4.508	1.000	-13.107	11.367
	Control < 5years	-1.613	4.582	1.000	-14.051	10.824

Control > 5 years	Autism < 5years	-11.763*	3.429	0.006	-21.071	-2.457
	Autism > 5 years	-2.483	3.244	1.000	-11.290	6.322
Autism < 5 years	Control < 5years	10.150	4.643	0.193	-2.452	22.752
	Control > 5 years	11.763*	3.429	0.006	2.457	21.071
	Autism > 5 years	9.280*	3.329	0.041	0.243	18.317
Autism > 5 years	Control < 5years	0.870	4.508	1.000	-11.367	13.107
	Control > 5 years	2.483	3.244	1.000	-6.322	11.290
	Autism < 5years	-9.280*	3.329	0.041	-18.317	-0.243

*. Mean difference is significant at $p < 0.05$

Table S9. Between-group ANOVA analysis for urinary glutamic acid values

	Sum of squares	df	Mean of squares	F	Sig.
Between groups	2284.947	3	761.649	2.917	0.040
Within groups	18538.333	71	261.103		

Table S10. Bonferroni correction for multiple comparisons by age between groups for urinary glutamic acid

(I)age groups	(j) age groups	Mean difference (I-J)	Standard error	Sig.	95% Confidence	
					Inferior limit	Superior limit
Control < 5 years	Control > 5years	4.715	6.671	1.000	-13.391	22.823
	Autism < 5 years	-3.375	6.759	1.000	-21.722	14.972
	Autism > 5years	10.505	6.563	0.684	-7.310	28.320
Control > 5 years	Control < 5 years	-4.715	6.671	1.000	-22.823	13.391
	Autism < 5 years	-8.090	4.992	0.657	-21.641	5.459
	Autism > 5years	5.789	4.723	1.000	-7.032	18.610
Autism < 5 years	Control < 5 years	3.375	6.759	1.000	-14.972	21.722
	Control > 5years	8.090	4.992	0.657	-5.459	21.641
	Autism > 5years	13.880*	4.847	0.033	0.723	27.037
Autism > 5years	Control < 5 years	-10.505	6.563	0.684	-28.320	7.310
	Control > 5years	-5.789	4.723	1.000	-18.610	7.032
	Autism < 5 years	-13.880*	4.847	0.033	-27.037	-0.723

*. Mean difference is significant at $p < 0.05$

Table S11. ANOVA test for the comparative analysis of urinary beta-alanine by age between the control group and the control group

	Sum of squares	df	Mean of squares	F	Sig.
Between groups	11847.906	3	3949.302	6.066	0.001
Within groups	46221.481	71	651.007		

Table S12. Tamhane test for multiple comparisons between urinary beta-alanine values according to age groups for the studied groups (autism and control)

(I)age groups	(j) age groups	Mean difference (I-J)	Standard error	Sig.	95% Confidence Inferior limit	95% Confidence Superior limit
Control < 5 years	Control > 5 years	12.636	11.821	0.893	-26.120	51.392
	Autism < 5 years	-12.950	13.518	0.927	-53.758	27.858
	Autism > 5 years	17.680	11.163	0.629	-21.152	56.512
Control > 5 years	Control < 5 years	-12.636	11.821	0.893	-51.392	26.120
	Autism < 5 years	-25.586	9.376	0.061	-51.933	0.760
	Autism > 5 years	5.043	5.458	0.933	-10.206	20.294
Autism < 5 years	Control < 5 years	12.950	13.518	0.927	-27.858	53.758
	Control > 5 years	25.586	9.376	0.061	-0.760	51.933
	Autism > 5 years	30.630*	8.532	0.009	6.107	55.153
Autism > 5 years	Control < 5 years	-17.680	11.163	0.629	-56.512	21.152
	Control > 5 years	-5.043	5.458	0.933	-20.294	10.206
	Autism < 5 years	-30.630*	8.532	0.009	-55.153	-6.107

*. Mean difference is significant at $p < 0.05$

Table S13. ANOVA test for comparison of urinary alpha-amino-adipic acid by age between controls and controls

	Sum of squares	df	Mean of squares	F	Sig.
Between groups	45577.639	3	15192.546	6.582	0.001
Within groups	163881.348	71	2308.188		

Table S14. Tamhane test for multiple comparisons by age group for urinary alpha aminoadipic acid.

(I)age groups	(j) age groups	Mean difference (I-J)	Standard error	Sig.	95% Confidence	
					Inferior limit	Superior limit
Control < 5 years	Control > 5 years	39.693	24.365	0.603	-45.156	124.543
	Autism < 5years	-16.925	28.466	0.993	-104.320	70.470
	Autism > 5 years	36.275	24.406	0.687	-48.540	121.090
Control > 5 years	Control < 5years	-39.693	24.365	0.603	-124.543	45.156
	Autism < 5years	-56.618*	16.870	0.016	-104.953	-8.284
	Autism > 5 years	-3.418	8.364	0.999	-26.436	19.600
Autism < 5 years	Control < 5years	16.925	28.466	0.993	-70.470	104.320
	Control > 5 years	56.618*	16.870	0.016	8.284	104.953
	Autism > 5 years	53.200*	16.930	0.026	4.755	101.645
Autism > 5 years	Control < 5years	-36.275	24.406	0.687	-121.090	48.540
	Control > 5 years	3.418	8.364	0.999	-19.600	26.436
	Autism < 5years	-53.200*	16.930	0.026	-101.645	-4.755

*. Mean difference is significant at $p < 0.05$

Table S15. Tamhane multiple comparisons test by age for urinary valine.

(I)age groups	(j) age groups	Mean difference (I-J)	Standard error	Sig.	95% Confidence	
					Inferior limit	Superior confidence
Control < 5years	Control > 5 years	13.670	11.040	0.821	-24.148	51.489
	Autism < 5years	-13.125	13.115	0.912	-52.816	26.566
	Autism > 5 years	10.705	11.007	0.930	-27.125	48.535
Control > 5 years	Control < 5years	-13.670	11.040	0.821	-51.489	24.148
	Autism < 5years	-26.795*	8.395	0.022	-50.749	-2.842
	Autism > 5 years	-2.965	4.429	0.986	-15.161	9.230

Autism < 5years	Control < 5years	13.125	13.115	0.912	-26.566	52.816
	Control > 5 years	26.795*	8.395	0.022	2.842	50.749
	Autism > 5 years	23.830	8.351	0.050	-.027	47.687
Autism > 5 years	Control < 5years	-10.705	11.007	0.930	-48.535	27.125
	Control > 5 years	2.965	4.429	0.986	-9.230	15.161
	Autism < 5years	-23.830	8.351	0.050	-47.687	0.027

*. Mean difference is significant at $p < 0.05$

Table S16. Tamhane test for multiple comparisons. according to age groups for proline in urine.

(I)age groups	(j) age groups	Mean difference (I-J)	Standard error	Sig.	95% Confidence	
					Inferior limit	Superior limit
Control < 5years	Control > 5 years	24.988	19.279	0.801	-44.646	94.624
	Autism < 5years	13.275	19.506	0.987	-56.063	82.613
	Autism > 5 years	23.765	19.304	0.833	-45.834	93.364
Control > 5 years	Control < 5years	-24.988	19.279	0.801	-94.624	44.646
	Autism < 5years	-11.713*	3.331	0.011	-21.286	-2.142
	Autism > 5 years	-1.223	1.807	0.985	-6.208	3.761
Autism < 5years	Control < 5years	-13.275	19.506	0.987	-82.613	56.063
	Control > 5 years	11.713*	3.331	0.011	2.142	21.286
	Autism > 5 years	10.490*	3.473	0.032	0.632	20.348
Autism > 5 years	Control < 5years	-23.765	19.304	0.833	-93.364	45.834
	Control > 5 years	1.223	1.807	0.985	-3.761	6.208
	Autism < 5years	-10.490*	3.473	0.032	-20.348	-0.632

*. Mean difference is significant at $p < 0.05$

Table S17. Age group Mann – Whitney for urinary methionine

Mann-Whitney U	125.500
Wilcoxon W	450.500
Z	-2.852
Asymp. Sig. (2-tailed)	0.004
a variables grouped by age groups	

Table S 18. Descriptive analysis for urinary sarcosine by age and Kruskal Wallis Test for multiple comparisons between groups.

	N	Mean	SD	Standard error	95% Confidence for the Mean		Minimum	Maximum
					Inferior limit	Superior limit		
Control < 5 years	8	2.625	1.187	0.419	1.632	3.618	2.0	5.0
Control > 5 years	22	2.136	0.639	0.136	1.853	2.420	2.0	5.0
Autism < 5years	20	3.050	1.538	0.343	2.330	3.770	2.0	6.0
Autism > 5 years	25	2.240	0.522	0.104	2.024	2.456	2.0	4.0

Statistical test[a,b] Sarcosine

Chi-square	8.493
df	3
Asymp. Sig.	0.037

a. Kruskal Wallis Test

b. Grouped variables: age groups

*. Mean difference is significant at $p < 0.05$

Table S19. Mann–Whitney test for sarcosine in urine

Test Statistics[a]	
Mann-Whitney U	142.500
Wilcoxon W	395.500
Z	-2.721
Asymp. Sig. (2-tailed)	0.007

a. Grouped variables: age groups

Table S20. Mann – Whitney Test for urinary alanine

Statistica Testului[a]	
Mann-Whitney U	126.000
Wilcoxon W	379.000
Z	-2.368
Asymp. Sig. (2-tailed)	0.018

a. Variabile grupate: grupe de vârstă
